# Evaluating The Effect of Botulinum Neurotoxin on the Improvement of Parafunctional and Dysfunctional Symptoms of the Temporomandibular Joint: A Pilot Study: Therapeutic Effect of Botulinum Neurotoxin on Temporomandibular Joint

**DOI:** 10.31661/gmj.v13iSP1.3650

**Published:** 2024-12-31

**Authors:** Abbas Haghighat, Mohammad Gholrokhian Thani

**Affiliations:** ^1^ Faculty of Dentistry, Isfahan University of Medical Sciences, Isfahan, Iran; ^2^ Department of Oral and Maxillofacial Surgery, Faculty of Dentistry, Isfahan University of Medical Sciences, Isfahan, Iran

**Keywords:** Temporomandibular Disorders (TMD), BoNT-A, Masseter Muscle, Bruxism

## Abstract

**Background:**

Temporomandibular disorders (TMD) are a wide range of clinical abnormalities affecting the masticatory muscles, the temporomandibular joint (TMJ), the surrounding bony structures, the soft tissues, or any combination of these components. Botulinum Neurotoxin Type A (BoNT-A) is one of the newest and most potent ways to slow or stop muscle activity. So this study aimed to evaluate the efficacy of BoNT-A injections in reducing symptoms of TMD.

**Materials and Methods:**

This pilot experimental study was conducted in the oral and maxillofacial surgery department of Isfahan University of Medical Sciences in 2022. Fifty patients with TMD were recruited. Participants received BoNT-A injections as the intervention. Outcomes measured included mouth opening, bite force, and joint pain/discomfort, assessed before and 4, 8, and 12 weeks after injection. Statistical analysis was performed using SPSS software.

**Results:**

This study included 24 male and 26 female participants, with a mean age of 29.92 ± 7.44 years. Chewing force significantly decreased four weeks after the injection compared to the baseline. From the fourth to the eighth week, the chewing force continued to decrease, but this was not statistically significant (P=0.820). Finally, chewing force gradually and significantly increased by the twelfth-week post-injection (P0.001), although it did not return to the pre-injection level. After the injection, pain-free mouth opening began to increase, which was not significant during the first four weeks (P=0.711) but became significantly greater in the eighth and twelfth weeks post-injection (P0.001In addition, compared to before the injection, pain and discomfort were significantly reduced four weeks later, according to the VAS test results. Furthermore, pain significantly decreased from week four to week eight (P0.001), and discomfort decreased steadily from week eight to week twelve. Nevertheless, the statistical significance of this decline was not established (P=0.132).

**Conclusion:**

Based on the results, BoNT-A injections reduce chewing force, alleviate pain and discomfort, and increase pain-free mouth opening. Therefore, BoNT-A has beneficial therapeutic effects on relieving symptoms of parafunction and dysfunction in the temporomandibular joint.

## Introduction

Temporomandibular disorders (TMD) are a large class of clinical conditions that can affect the masticatory muscles, the temporomandibular joint (TMJ), the surrounding bone structures, the soft tissues, or any combination of these parts. Symptoms of TMD include a loss of range of motion in the lower jaw, pain in the joint, restricted or abnormal jaw opening, and stiffness in the masticatory muscles [1]. Jaw clenching, grinding, and/or thrusting are the hallmarks of bruxism, a repetitive masticatory muscle activity that can occur while awake or asleep. There are several factors that can contribute to this issue, including anxiety, stress, depression, personality types, nutritional deficiencies (such as magnesium, calcium, iodine, and vitamin complexes), poor dental occlusion, disorders affecting the central nervous system, certain medications, lack of oral proprioception, and genetics [3]. While several factors have been postulated as potential causes of bruxism, including emotional stress, neurological disorders, specific medications, and occlusal interferences, the exact origins and pathophysiology of the disorder remain a mystery [4]. Because bruxism can lead to tooth fractures, denture wear and loosening, temporomandibular joint difficulties, masticatory muscle soreness, fatigue, and masseter muscle hypertrophy, controlling the habit is essential for reducing its negative effects [5][6][7][8]. The masseter, temporalis, and medial pterygoid muscles are involved in chewing and that are responsible for closing the lower jaw. The force that is generated when chewing is mostly generated by these muscles. The jaw-closing movement, which increases the total chewing force, is provided by the three muscles working together. The masseter muscle alone is responsible for producing around 43% of the total chewing force [9][10][11]. The masseter muscle in particular can enlarge as a consequence of chronic bruxism. Masseter hypertrophy [12] is a growing growth of the masseter muscle that is asymptomatic and can occur on one or both sides of the body. Although it is less prevalent, unilateral hypertrophy can nonetheless occur based on how someone chews. Consequently, face enlargement and an angular appearance may be symptoms of this condition [13]. Many different methods, including medication, physical therapy, and dental procedures, have been suggested for the treatment or management of joint-related issues. The most common approaches include the use of both occlusal splints and systemic pharmaceuticals, such as anti-inflammatory, antidepressant, anxiety, or muscle relaxant drugs [14]. These conventional treatments may not work 100% of the time, thus more research into complementary and alternative medicine is required.

Exotoxin BTX-A, generated by the bacterium Clostridium botulinum, reduces or inhibits muscle action by blocking the release of acetylcholine from cholinergic nerve terminals at the neuromuscular junction. The US Food and Drug Administration (FDA) has given the green light to BTX-A for a variety of uses, including alleviating symptoms of cervical dystonia, severe primary axillary hyperhidrosis, strabismus, blepharospasm, hemifacial spasm, and glabellar wrinkles [15][16][17]. To get the most out of this treatment, you need to pick the active muscles correctly [18]. A thorough understanding of the lower face’s anatomy, muscle interactions, aesthetics, and the risks of injecting botulinum neurotoxin in the wrong places is necessary for a successful procedure [19]. In order to avoid treatment-related problems, it is crucial to do a clinical evaluation of vital signs, apply the correct dosage, and follow all pre- and post-treatment instructions [20]. Recent research has looked at the results of injecting botulinum neurotoxin into the masseter muscle. To minimize unwanted side effects, it is essential to inject botulinum neurotoxin only into the targeted muscle area when injecting it into the masseter [21]. Chen and colleagues [22] analyzed research that demonstrated several points at which botulinum injections were administered; some studies used a two-point injection approach, while others used a three-point or five-point injection method, each with its own set of specifics. Rathod et al. [21] proposed a 6-point injection technique for botulinum toxin injection into the masseter muscle; Although this study was a case report, the results suggest that the 6-point injection technique may be an effective and patient-satisfactory approach for treating masseter muscle hypertrophy. Given the existing research on the efficacy of BoNT-A injections in the masseter muscle, and considering the high prevalence and significant impact of TMD symptoms on patients’ quality of life, it is essential to investigate the effects of BoNT-A injections on TMD symptoms. Therefore, this study aims to compare the chewing force, pain, and maximum mouth opening of patients experiencing TMD symptoms before and after receiving injections of BoNT-A.

## Materials and Methods

### Study Design, Settings, Population, and Ethical Approval

This pilot experimental study was approved by the Institutional Review Board (IRB) under the code IR.MUI.DHMT.REC.1402.105. The study was conducted at the oral and maxillofacial surgery department, and a total of 50 patients undergoing treatment for TMD were recruited.

### Inclusion and Exclusion Criteria

The inclusion criteria for this study were carefully defined to ensure that only patients with moderate to severe TMD symptoms were included. The criteria were as follows: [1] patients exhibiting symptoms of TMD as determined by a clinical examination and questionnaire, [2] patients aged between 18 and 55 years, and [3] patients with moderate to severe discomfort in the masticatory muscles. These criteria were established to ensure that the study population was homogeneous and that the results would be generalizable to patients with similar characteristics.

In contrast, the exclusion criteria were designed to eliminate patients who may have had conditions that could affect the study outcomes or compromise the safety of the participants. The exclusion criteria included [1] patients who refused to allow the research project to use their data, [2] patients with loss of two or more posterior teeth (excluding the third molar), [3] patients with fixed or removable prostheses involving more than four dental units, [4] patients with advanced malocclusion, [5] known allergy to Botox, [6] pregnancy, [7] neuromuscular function conditions, [8] bleeding disorders, [9] infectious lesions in the injection area, and [10] patients with nighttime coughs. By excluding these patients, we aimed to minimize any potential confounding variables that could affect the study outcomes.

### Data Collection and Measurements

The following characteristics were evaluated in this study: [1] mouth opening, [2] bite force, and [3] joint pain and discomfort. Mouth opening was measured before and after surgery using a digital caliper. Patients were asked to open their mouths as wide as they could without hurting themselves. Bite force was measured using the MES Bite Force Meter (TCS-1T) available at the Research Center of the School of Dentistry, Isfahan University of Medical Sciences. The bite force was measured at teeth 11 on the upper jaw and tooth 41 on the lower jaw. Joint pain and discomfort were quantified using the Visual Analog Scale (VAS), a horizontal scale from 0 cm (no pain) to 10 cm (worst anguish).

### Follow-Up Evaluations

Follow-up evaluations were conducted at three time points: Week 4 Post-Injection, Week 8 Post-Injection, and Week 12 Post-Injection. At each follow-up visit, patients underwent the same measurements as at baseline.

### Statistical Analysis

Statistical analysis was performed using SPSS software version 26. The following tests were used: [1] Shapiro-Wilk test to check for normality of data distribution, [2] Mauchly’s Sphericity Test to check for sphericity assumption, and [3] Pairwise Comparisons with Bonferroni adjustments to compare the means of different groups. P-values of under 0.05 were considered statistically significant.

## Results

**Table:** Table1. Distribution of Participants Based on Demographic Variables

**Variable**	**Unit**	**Mean ± SD/ Frequency (%)**
**Age**	**Years**	29.92 ± 7.44
**Gender**	**Male**	24 (48.0%)
	**Female**	26 (52.0%)

**Table:** Table2. Assessment and comparison of patient’s bite force¸ Pain-Free Mouth Opening, and VAS before injection, four weeks, eight weeks, and twelve weeks after injection of BoNT-A (in newtons)

**Test Performed **	**Before Injection **	**Week 4 Post-Injection **	**Week 8 Post-Injection **	**Week 12 Post-Injection **	**p-value (week 4 vs Before) **	**p-value (Week 8 vs Week 4) **	**p-value (Week 12 vs Week 8) **
**Patient Bite Force Test (N) **	69.64±8.07	36.46±4.22	36.08±4.47	59.60±16.19	<0.001	0.820	<0.001
**Pain-Free Mouth Opening (mm) **	30.42±2.11	30.48±1.65	32.20±1.76	33.04±1.64	0.711	<0.001	<0.001
**VAS (pain and discomfort) **	6.24±0.91	3.10±0.58	3.60±0.63	3.82±0.77	<0.001	<0.001	0.132

Participants in this trial were 26 females and 24 males who were all receiving therapy with BoNT-A. According to Table-1, the participants’ average age was 29.92 ± 7.44 years.

Before, four, eight, and twelve weeks following injection of BoNT-A, the patient’s bite force was assessed in Newtons, as shown in Table-2. After four weeks of injection, the bite force was much lower than it had been beforehand. Bite force also decreased between weeks 4 and 8 following injection, however this trend was not statistically significant (P = 0.820). The biting force gradually and considerably rose (P < 0.001) by the twelfth week after injection, although it did not go back to its initial levels before injection.

As observed, following the BoNT-A injection, the pain-free mouth opening increased progressively. However, this increase was not statistically significant in the first four weeks post-injection (P = 0.711). Significant improvements were noted in the eighth and twelfth weeks post-injection (P < 0.001).

The patient’s reported pain and suffering is significantly reduced four weeks after injection compared to before treatment. In addition, there is a continuous and significant reduction in pain levels (P < 0.001) from week four to week eight following the injection. In conclusion, pain levels do go down from week 8 to week 12 after injection, however it’s not a statistically significant drop (P = 0.132).

## Discussion

This study aimed to evaluate the effectiveness of a specific treatment approach on TMD symptoms and identify any significant changes in mouth opening, bite force, and joint pain and discomfort. In our study, chewing force was shown to be considerably lower four weeks following injection of BoNT-A compared to levels before injection. Furthermore, between weeks 4 and 8, chewing force decreased, however this change was not statistically significant (P = 0.820). Chewing force increased gradually and significantly (P < 0.001) by the twelfth week post-injection, although it did not go back to its initial values recorded before the injection. In two separate studies, Swan et al. [23] and Zhang et al. [24] discovered that the maximal occlusal force was significantly different in the BTX-A group compared to the control and placebo groups. However, in neither of these studies were there significant changes between the control and placebo groups. Results from the study by Sewane et al. indicate that the maximal occlusal force in the BTX-A group changed significantly with time, reaching its lowest point three months after treatment [23]. The occlusal force was also shown to be statistically lower six months after treatment ended compared to levels before treatment. Regardless of the study group, Ramalho et al. [25] found that patients who underwent evaluation showed less biting force and orofacial pain. Our work, together with that of Ramalho et al., and other investigations, provide credence to these conclusions, indicating that BTX-A is a viable approach for treating bruxism by lowering chewing power. These findings are supported by research conducted by Ægren et al. (2020) and Muñoz (2019) [26][27]. Also, Muñoz and Ågren found that biting force rebounded to near baseline levels 120 days after the reduction of pain-related chemicals, such as substance P, compared to 180 days for pain levels, mostly because of presynaptic mediators [28]. Regardless, treatment satisfaction was excellent in both groups throughout all assessment times. The researchers also noted a marked reduction in bruxism, a condition that lowers quality of life due to tooth wear, disruption of vital dental functions, sleep problems, and overall disruption [29]. Hence, BTX-A could be quite useful as it doesn’t depend on patients following instructions like physiotherapy and occlusal splints do [26].

A painless assessment of the patient’s mouth openness revealed that following the injection of BoNT-A, it had increased. However, after the first four weeks of injection, there was no statistically significant increase (P = 0.711). The eighth and twelfth weeks after injection, nevertheless, showed considerable improvements. The masticatory muscles that are nearby can be relaxed, inflammation reduced, and mouth opening improved with BoNT/A treatment, according to previous research [30][31]. Therefore, the mandible’s range of motion can be used to measure BoNT/A’s treatment efficacy. Multiple studies have shown that after receiving BoNT injections, patients’ ability to open their mouths is significantly improved, allowing them to open their mouths a lot wider. Consistent with our results, Dela Torre Canales et al. [32] just published a study that found that after 180 days, independent of the dose, the BoNT/A group had significantly better mouth opening than the saline injection group (P < 0.05). The maximal mouth-opening range was shown to be significantly higher in the BoNT/A group compared to the saline placebo group at baseline, one week, one month, and six months after injection, according to Guarda-Nardini et al. [33][34]. However, three months after injection, the maximal mouth-opening range was significantly higher in the saline placebo group, according to a research by Nijdam et al. Alternatively, maximum mouth opening was found to be reduced in the BoNT group [35].

Based on the evaluation of pain and discomfort in the joint area using the VAS test, the study found that four weeks following the injection of BoNT-A, the patient reported much less pain and discomfort than previously. Furthermore, from week four to week eight after the injection, the patient reported significantly less pain. From the ninth to the twelfth week after the injection, the discomfort eventually subsided. But this decline was not statisticaly significant.

The visual analog scale has been the standard assessment tool in most studies that have used pain as a primary criterion. However, Kurtoglu et al. [36] used other diagnostic criteria, specifically the Research Diagnostic Criteria for Temporomandibular Disorders (RDC/TMD) Axis II, to evaluate the severity of pain. Ernberg et al. [37] found that BTX-A reduced pain more effectively than saline at a one-month follow-up. The average reduction in pain following BTX-A was 30% on a VAS scale from 0 to 100, while the average reduction following saline was 11%. There was a 23% decrease in pain in the BTX-A group and a 4% decrease in the saline group at the three-month follow-up. In addition, the study by Nardini et al. [33] found that BTX reduced resting pain from an initial score of 5.00 ± 3.62 to 3.60 ± 2.88, while the placebo group experienced a decrease from 3.90 ± 2.92 to 4.10 ± 2.58 over the course of two months. In addition, after six months, the BTX group reported less pain when chewing, going from an initial score of 6.20 ± 2.78 to 2.37 ± 3.60. In comparison, the placebo group went from an initial score of 4.92 ± 2.10 to 2.79 ± 3.70.

Compared to the placebo group, those who received Botox showed statistically significant pain reduction at baseline, one week, one month, and six months after treatment. The authors of the study by Kim et al. [38] found that the BTX group experienced higher pain scores than the placebo group, especially when it came to the intensity of facial pain (OVAS), and this difference became even more noticeable at four weeks. The OVAS groups did not differ significantly from one another, nevertheless, when comparing BTX to placebo. The BoNT-A group benefited from the change in VAS for pain, according to Ondo et al. [39] who used a 2-tailed T-test. At four weeks, the IncobotulinumtoxinA group reported far less discomfort than the placebo group, according to the research by Patel et al. [40]. The BTX-A group reported less pain both at rest and when chewing, according to Sewane et al. [23]. On the other hand, there was no discernible difference in pain levels between the control and placebo groups.

On average, compared to the placebo group, 91% of BTX patients reported a 3.2 reduction in VAS pain, according to Lindern et al. [41]. It was also observed that 26 patients with baseline pain levels of 6.5 or higher shown significant improvement (≥ 3.5), while 27 patients with baseline pain scores of less than 6.5 exhibited only moderate improvement (≤ 3.5). Researchers Kurtoglu et al. [36] found that BTX reduced pain levels. Nixdorf et al. [35] found that the masseter muscular action potential decreased on day 14, then increased on day 28 of their investigation. But even on day 28, the pain scores kept going down. There was no statistically significant difference in pain intensity between the BTX and placebo groups, according to these researchers [35]. Parafunction and temporomandibular dysfunction patients often feel oral and facial pain, which is most likely caused by hyperactive muscles [26]. Because BTX-A causes temporary paralysis or changes neuromuscular contraction, it is possible that it will relax muscles and reduce discomfort [ 42]. Furthermore, BTX-A may exert its analgesic effects via two additional pathways: [1] reducing the number of transient receptor potential channels on the membrane of neuronal cells and [2] inhibiting the release of pain-related neurotransmitters (such as substance P from dorsal root ganglia) [42]. The goals of our investigation were similar to those of Song et al. [34] who looked at BoNT-As a potential treatment for TMD. Their goal was to create and specify an algorithm for the treatment of TMD. Prior to administering BTX-A injections, the researchers recommended conservative treatments including behavioral therapy, oral devices, warm compresses, and medicine (including anti-inflammatory and muscle relaxant pharmaceuticals). Hence, BTX-A should only be administered to individuals who have not shown improvement with more conservative therapy options. For patients with TMD, they also proposed a BTX-A dosage: 7.5-10 units for the lateral pterygoid muscles (LP) in instances of submaxillary discomfort, jaw deviation, or behaviors like bruxism [34].

### Limmitations of study

This pilot study has several limitations that should be considered when interpreting its results. Firstly, the sample size is relatively small, with only 50 participants, which may not be representative of the larger population of individuals with temporomandibular disorders. Furthermore, the study’s design is a pre-post study, which lacks a control group, making it difficult to attribute the observed effects solely to the BoNT-A injections. These limitations show the need for a larger, randomized controlled trial with a longer follow-up period to confirm the findings and establish the efficacy and safety of BoNT-A injections for treating TMD.

## Conclusion

The results of this study show that patients experience less masticatory force, less pain and discomfort, and a greater range of painless mouth opening after receiving an injection of BoNT-A. This pilot study provides preliminary evidence that Botulinum Neurotoxin Type A injections may have an impact on symptoms of temporomandibular disorders. The observed changes in chewing force, pain-free mouth opening, and pain/discomfort over time suggest that BoNT-A injections may be worth further investigation as a potential treatment option for TMD.

## Conflict of Interest

There are no conflicts of interest to declare.

## References

[R1] Wadhwa S, Kapila S (2008). TMJ disorders: future innovations in diagnostics and
therapeutics. Journal of dental education.

[R2] De la, Câmara-Souza MB, Do Amaral, Garcia RC, Manfredini D (2017). Is there enough evidence to use botulinum toxin injections for
bruxism management A systematic literature review. Clinical oral investigations.

[R3] Sposito MM (2014). Teixeira SAF: Botulinum toxin A for bruxism: a systematic
review. Acta Fisiatr.

[R4] Beddis H, Davies S (2018). Sleep bruxism: an overview for clinicians. Br Dent J.

[R5] Johansson A, Omar R, Carlsson GE (2011). Bruxism and prosthetic treatment: a critical review. Journal of prosthodontic research.

[R6] Glaros AG (J). Tooth contact versus clenching: Oral parafunctions and facial
pain.

[R7] Okeson JP (2013). Etiology of functional disturbances in the masticatory system. Management of temporomandibular disorders and occlusion.

[R8] Carlsson GE, Egermark I, Magnusson T (2003). Predictors of bruxism, other oral parafunctions, and tooth wear
over a 20-year follow-up period. J Orofac Pain.

[R9] Weijs WA, Hillen B (1985). Cross-sectional areas and estimated intrinsic strength of the
human jaw muscles. Acta morphologica neerlando-scandinavica.

[R10] Peck CC (2016). Biomechanics of occlusion–implications for oral rehabilitation. Journal of oral rehabilitation.

[R11] Ingawalé SM GT (2012). Biomechanic of the temporomandibular joint. 1st ed ed. London: InTech.

[R12] Erdil D, Bagis N, Eren H, Camgoz M, Orhan K (2023). The evaluation of the relationship between changes in masseter
muscle thickness and tooth clenching habits of bruxism patients treated with
botulinum toxin A. Journal of Medical Ultrasound.

[R13] Aguilera SB, Perico VA (2017). Aesthetic treatment of bruxism. J Clin Aesthet Dermatol.

[R14] Yurttutan ME, Sancak KT, Tüzüner AM (2019). Which treatment is effective for bruxism: occlusal splints or
botulinum toxin.. Journal of Oral and Maxillofacial Surgery.

[R15] Lang A (2004). History and uses of BOTOX®(botulinum toxin type A). Professional Case Management.

[R16] Rauso R, Tartaro G, Zerbinati N, Nicoletti GF, Fragola R (J). Botulinum toxin type A injections for masticatory muscles
hypertrophy: A systematic review.

[R17] D’Souza A (2020). Applied anatomy for botulinum toxin injection in cosmetic
interventions. Curr Otorhinolaryngol Rep.

[R18] Carruthers J (Dermatol). Botulinum toxin A in the mid and lower face and neck.

[R19] Coleman KR, Carruthers J (2006). Combination therapy with BOTOX™ and fillers: the new rejuvnation
paradigm. Dermatologic therapy.

[R20] Rathod NN, John RS (2023). Botulinum toxin injection for masseteric hypertrophy using 6
point injection technique–a case report Proposal of a clinical technique to
quantify prognosis. Clinical, Cosmetic and Investigational Dentistry.

[R21] Chen Y, Tsai C-H, Bae TH, Huang C-Y, Chen C, Kang Y-N, et al (2023). Effectiveness of botulinum toxin injection on bruxism: a
systematic review and meta-analysis of randomized controlled trials. Aesthetic plastic surgery.

[R22] Jadhao VA, Lokhande N, Habbu SG, Sewane S, Dongare S, Goyal N (2017). Efficacy of botulinum toxin in treating myofascial pain and
occlusal force characteristics of masticatory muscles in bruxism. Indian Journal of Dental Research.

[R23] Zhang L-d, Liu Q, Zou D-r, Yu L-f (2016). Occlusal force characteristics of masseteric muscles after
intramuscular injection of botulinum toxin A (BTX–A) for treatment of
temporomandibular disorder. British Journal of Oral and Maxillofacial Surgery.

[R24] da Silva, Palma LF, Ramalho KM, Tedesco TK, Morimoto S (2023). Effect of botulinum toxin A on pain, bite force, and satisfaction
of patients with bruxism: A randomized single-blind clinical trial comparing
two protocols. The Saudi Dental Journal.

[R25] Ågren M, Sahin C, Pettersson M (2020). The effect of botulinum toxin injections on bruxism: A systematic
review. Journal of oral rehabilitation.

[R26] Muñoz Lora, Del Bel, Jabbari B, Lacković Z (2019). Botulinum toxin type A in dental medicine. Journal of dental research.

[R27] Kwon KH, Shin KS, Yeon SH, Kwon DG (2019). Application of botulinum toxin in maxillofacial field: Part II
Wrinkle, intraoral ulcer, and cranio-maxillofacial pain. Maxillofacial plastic and reconstructive surgery.

[R28] Lobbezoo F, Ahlberg J, Raphael K, Wetselaar P, Glaros A, Kato T, et al (2018). International consensus on the assessment of bruxism: Report of a
work in progress. Journal of oral rehabilitation.

[R29] Thambar S, Kulkarni S, Armstrong S, Nikolarakos D (2020). Botulinum toxin in the management of temporomandibular disorders:
a systematic review. British Journal of Oral and Maxillofacial Surgery.

[R30] Freund B, Schwartz M, Symington JM (1999). The use of botulinum toxin for the treatment of temporomandibular
disorders: preliminary findings. Journal of oral and maxillofacial surgery.

[R31] De la, Poluha RL, Pinzón NA, Da Silva, Almeida AM, Ernberg M, et al (2022). Efficacy of botulinum toxin type-A I in the improvement of
mandibular motion and muscle sensibility in myofascial pain TMD Subjects: A
randomized controlled trial. Toxins.

[R32] Guarda-Nardini L, Manfredini D, Salamone M, Salmaso L, Tonello S, Ferronato G (2008). Efficacy of botulinum toxin in treating myofascial pain in
bruxers: a controlled placebo pilot study. CRANIO®.

[R33] Song P, Schwartz J, Blitzer A (2007). The emerging role of botulinum toxin in the treatment of
temporomandibular disorders. Oral diseases.

[R34] Nixdorf DR, Heo G, Major PW (2002). Randomized controlled trial of botulinum toxin A for chronic
myogenous orofacial pain. Pain.

[R35] Kurtoglu C, Gur OH, Kurkcu M, Sertdemir Y, Guler-Uysal F, Uysal H (2008). Effect of botulinum toxin-A in myofascial pain patients with or
without functional disc displacement. Journal of oral and maxillofacial surgery.

[R36] Ernberg M, Hedenberg-Magnusson B, List T, Svensson P (2011). Efficacy of botulinum toxin type A for treatment of persistent
myofascial TMD pain: a randomized, controlled, double-blind multicenter
study. Pain.

[R37] Kim SR, Chang M, Kim AH, Kim ST (2023). Effect of Botulinum Toxin on Masticatory Muscle Pain in Patients
with Temporomandibular Disorders: A Randomized, Double-Blind,
Placebo-Controlled Pilot Study. Toxins.

[R38] Ondo WG, Simmons JH, Shahid MH, Hashem V, Hunter C, Jankovic J (2018). Onabotulinum toxin-A injections for sleep bruxism: A
double-blind, placebo-controlled study. Neurology.

[R39] Patel AA, Lerner MZ, Blitzer A (2017). IncobotulinumtoxinA injection for temporomandibular joint
disorder: A randomized controlled pilot study. Annals of Otology, Rhinology & Laryngology.

[R40] Von Lindern, Niederhagen B, Bergé S, Appel T (2003). Type A botulinum toxin in the treatment of chronic facial pain
associated with masticatory hyperactivity. Journal of oral and maxillofacial surgery.

[R41] Cheng Y, Yuan L, Ma L, Pang F, Qu X, Zhang A (2022). Efficacy of botulinum-A for nocturnal bruxism pain and the
occurrence of bruxism events: a meta-analysis and systematic review. British Journal of Oral and Maxillofacial Surgery.

